# Beyond access to medication: the role of SUS and the characteristics of HIV care in Brazil

**DOI:** 10.11606/s1518-8787.2023057004476

**Published:** 2023-08-28

**Authors:** Ana Maroso Alves, Angélica Carreira dos Santos, Aline Kumow, Ana Paula Sayuri Sato, Ernani Tiaraju de Santa Helena, Maria Ines Battistella Nemes

**Affiliations:** I Universidade de São Paulo Faculdade de Medicina Departamento de Medicina Preventiva São Paulo SP Brasil Universidade de São Paulo. Faculdade de Medicina. Departamento de Medicina Preventiva. São Paulo, SP, Brasil; II Universidade de São Paulo Faculdade de Medicina Departamento de Pediatria São Paulo SP Brasil Universidade de São Paulo. Faculdade de Medicina. Departamento de Pediatria. São Paulo, SP, Brasil; III Universidade de São Paulo Faculdade de Saúde Pública Departamento de Epidemiologia São Paulo SP Brasil Universidade de São Paulo. Faculdade de Saúde Pública. Departamento de Epidemiologia. São Paulo, SP, Brasil; IV Universidade de São Paulo Faculdade de Saúde Pública Departamento de Saúde Pública São Paulo SP Brasil Universidade de São Paulo. Faculdade de Saúde Pública. Departamento de Saúde Pública. São Paulo, SP, Brasil; V Universidade Regional de Blumenau Departamento de Medicina Blumenau SC Brasil Universidade Regional de Blumenau. Departamento de Medicina. Blumenau, SC, Brasil

**Keywords:** Anti-HIV Agents, supply & distribution, Health Care Quality, Access, and Evaluation, Unified Health System, Supplemental Health

## Abstract

**OBJECTIVE:**

To estimate the public-private composition of HIV care in Brazil and the organizational profile of the extensive network of public healthcare facilities.

**METHODS:**

Data from the Qualiaids-BR Cohort were used, which gathers data from national systems of clinical and laboratory information on people aged 15 years or older with the first dispensation of antiretroviral therapy between 2015–2018, and information from SUS healthcare facilities for clinical-laboratory follow-up of HIV, produced by the Qualiaids survey. The follow-up system was defined by the number of viral load tests requested by any SUS healthcare facility: follow-up in the private system – no record; follow-up at SUS – two or more records; undefined follow-up – one record. SUS healthcare facilities were characterized as outpatient clinics, primary care and prison system, according to the respondents’ self-classification in the Qualiaids survey (72.9%); for non-respondents (27.1%) the classification was based on the terms present in the names of the healthcare facilities.

**RESULTS:**

During the period, 238,599 people aged 15 years or older started antiretroviral therapy in Brazil, of which 69% were followed-up at SUS, 21.7% in the private system and 9.3% had an undefined system. Among those followed-up at SUS, 93.4% received care in outpatient clinics, 5% in primary care facilities and 1% in the prison system.

**CONCLUSION:**

In Brazil, antiretroviral treatment is provided exclusively by SUS, which is also responsible for clinical and laboratory follow-up for most people in outpatient clinics. The study was only possible because SUS maintains records and public information about HIV care. There is no data available for the private system.

## INTRODUCTION

From the introduction of AZT (zidovudine) in 1993 to the emergence of current highly active antiretroviral therapy (HAART), free and universal access to antiretroviral therapy for HIV/AIDS (ART) internationally distinguished the Brazilian response to the AIDS epidemic^1^ .

The institution of the national STD/AIDS program, in 1986^1^ , gave rise to the implementation of hospital and outpatient facilities to care for people with AIDS, most of them in pre-existing structures of the public health system^2^ , which, in 1988, became officially the Unified Health System (SUS)^3^ . The modality of outpatient clinical assistance, initially called a specialized healthcare facility for STD/AIDS, was implemented in several states of the country. With the spread of the epidemic in the country and the incorporation of antiretroviral therapy, which converted HIV into a chronic condition, the number of outpatients clinics expanded greatly: Ministry of Health records show that the number of facilities increased by more than 3,000% in 20 years (from 33 in 1996^4^ to 1,060 in 2016)^5^ .

As the structure of SUS is decentralized, the implementation of facilities became the responsibility of the municipalities. Serial surveys on the healthcare facilities’ organization showed that this type of management resulted in a heterogeneous set of facilities, with variable administrative configuration (primary care facilities, specialty clinics, specialized healthcare facilities for STI/HIV, hospital outpatient clinics) and volume of patients (from one to more than a thousand)^6^ .

Since the beginning of the implementation of drug treatment for HIV until today, SUS has been the only buyer and supplier of antiretroviral therapy drugs in Brazil, and medication is provided by public system services. Thus, people with a medical prescription for antiretrovirals are registered with a local SUS facility to receive the drugs. A national system continuously records all therapy dispensations (SICLOM – Medication Logistic Control System). In addition to this system, all viral load and CD4 tests performed in SUS are registered in a nationally centralized information system (SISCEL – Laboratory Test Control System). There are no public records that allow the monitoring of care follow-up by private facilities, although it is recommended that patients in the private system show the result of the most recent viral load test at the time of dispensing medication.

So far, there are no studies that outline the national organizational characteristics of HIV care in the country. Aiming to contribute to the improvement of service implementation policies, this study aims to estimate the public-private composition of HIV care in Brazil, as well as the organizational profile of the extensive network of public healthcare facilities.

## METHODS

### Data Source and Population

The study uses secondary and anonymized data from the ongoing research project “Coorte Qualiaids-BR”, approved by the Ethics Committee for Research with Human Beings (CAEE: 27659220.3.0000.0065), which gathers information from people aged 15 years or older with registration in the SICLOM of the first dispensation of antiretroviral therapy between January 1, 2015 and December 31, 2018, and information from outpatient HIV care facilities of SUS.

**Figure 1:**
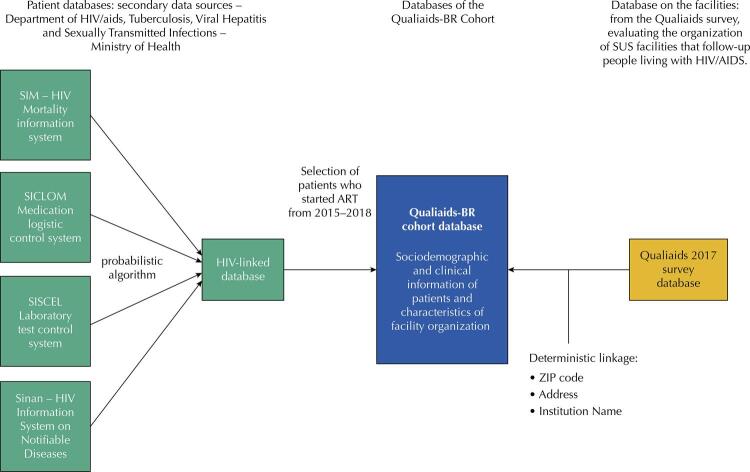
Data sources and construction process of the Qualiaids-BR Cohort database.

The Qualiaids-BR cohort database was built from two databases: 1) Database of people on antiretroviral therapy with clinical and sociodemographic data linked individually via a probabilistic algorithm, already validated and routinely used in the epidemiological bulletins of the Ministry of Health^9 , 10^ and in publications in the area^11 , 12^ . The database, produced annually by the ministry, lists, for each person on antiretroviral therapy, data from the SUS information systems – SICLOM, SISCEL, SINAN (Information System on Notifiable Diseases) and SIM (Information System on Mortality); 2) Database of healthcare facilities from the response to the Qualiaids 2016/17 survey on the organization of SUS facilities that prescribe antiretroviral drugs^5^ . The deterministic linkage between the two databases were based on location data (zip code, address) and facility names ( Figure 1 ).

### Definition of the Predominant Health Care System for Clinical Follow-up

To define the health system in which HIV patients are followed-up, we considered all the viral load tests requested during the treatment and the date of initiation of the antiretroviral therapy, according to the criteria summarized in Chart 1 .

**Chart 1:** Definition of the predominant follow-up system, according to the number of records of viral load (VL) tests and initiation of antiretroviral therapy (ART). Qualiaids-BR cohort, 2021.

Predominant follow-up system	Definition
SUS	**People with two or more VL records People with more than one VL record** – loss to follow-up^a^ within 66 days^b^ from starting ART
Undefined	**People without any VL record** – loss to follow-up within 66 days from starting ART **People with one VL record** – loss to follow-up after 66 days from starting ART **People without any record or with one VL record** – starting ART after 10/26/2018^c^
Private	**People without any record or with one VL record** – starting ART prior to 10/26/2018 **People without any record** – with loss to follow-up after 66 days of starting ART

^a^ Loss to follow-up was defined as death or definitive abandonment of ART (which considers whether the last medication withdrawal was recorded up to 100 days before the final date of the cohort: 12/31/2018), in line with the criteria defined by the Ministry of Health^17^.

^b^ The 66-day interval is based on the definition in the Clinical and Therapeutic Guidelines Protocol^27^ which states that, for people starting ART, the interval between VL exams should be up to 56 days. Another 10 days were added to this interval, including weekends and holidays.

^c^ The date was defined considering the end of the cohort (12/31/2018) and subtracted of 66 days.

For people predominantly followed-up at SUS healthcare facilities, the follow-up facility was defined as the one that requested the viral load tests. For those who had exams requested by more than one facility, the one that requested more exams was considered and, when the number was equal, the one with which the patient was engaged for longer was considered. The duration of engagement with the healthcare facility was calculated by the difference between the first and last request for viral load in the given facility.

To characterize the type of HIV care facility, the facility’s response to a single-answer structured question from the Qualiaids-2016/2017 survey^5^ was used. The alternatives describe the administrative types, and were grouped into two types: (1) Outpatient clinic (exclusive outpatient clinic for specialized care for patients with HIV/AIDS, STD and viral hepatitis outpatient clinic; Outpatient clinic specialized in infectious diseases; Specialized care team inserted in a primary care service; Outpatient care for various specialties and hospital outpatient clinic); (2) Primary care facility (community health center, family health center). For the facilities that did not respond to the survey, the attribution was made from the search for terms present in the facility registration name in SISCEL, as shown in Chart 2 .

**Chart 2:** Classification of types of healthcare facilities based on terms present in the name of the facility. Qualiaids-BR cohort, 2015–2018.

Type of facility	Name/terms
Outpatient clinic	AME (Specialty Medical Outpatient Clinic); Healthcare center for infectious and contagious diseases; Center specializing in infectious and contagious diseases; SAE (Specialized HIV care facility); Specialty Center; Diagnosis and Treatment Center; Guidance and Counseling Center; Reference Center; Testing and Counseling Center; Specialized Healthcare Center; Regional State Center of Medium and High Complexity; Regional Center of Specialties; Outpatient Complex; Polyclinic and Reference Unit
Primary care	Health Center; Municipal Health Center (CMS); Family Clinic (CF); Health Center (CS); Family Health Strategy (ESF); Family Health Support Center (NASF); Health Center; Community Health Center (UBS) and Health Unit
Prison system facility	House of Custody; Penitentiary; Penitentiary system; Prison; Center for assistance to imprisoned women; ‘Detention’ and ‘Criminal’
Could not be assigned	Health Support Center; Epidemiological Surveillance Unit; ‘Foundation’; Regional Health; Association; Support House; Department of Health Actions; Regional Board of Health and Intermunicipal Consortium

### Data Analysis

The absolute and relative distributions of people in the Qualiaids-BR Cohort were described, according to the predominant HIV follow-up system (SUS, private or undefined), Brazilian geographic state and type of SUS facilities (outpatient clinic, primary care and prison system).

## RESULTS

### Clinical-Laboratory Follow-up System

In the analyzed period, 238,599 people aged 15 years or older started antiretroviral therapy in Brazil. Of this total, 164,667 (69%) had clinical and laboratory follow-up in SUS facilities and 51,879 (21.7%) were followed up in private facilities. It was not possible to assign the predominant follow-up system for 22,053 (9.3%) people. Among people that received care at SUS, 132,086 (80.2%) had all the exams requested by the same facility and 32,581 (19.8%) by more than one facility.

**Figure 2:**
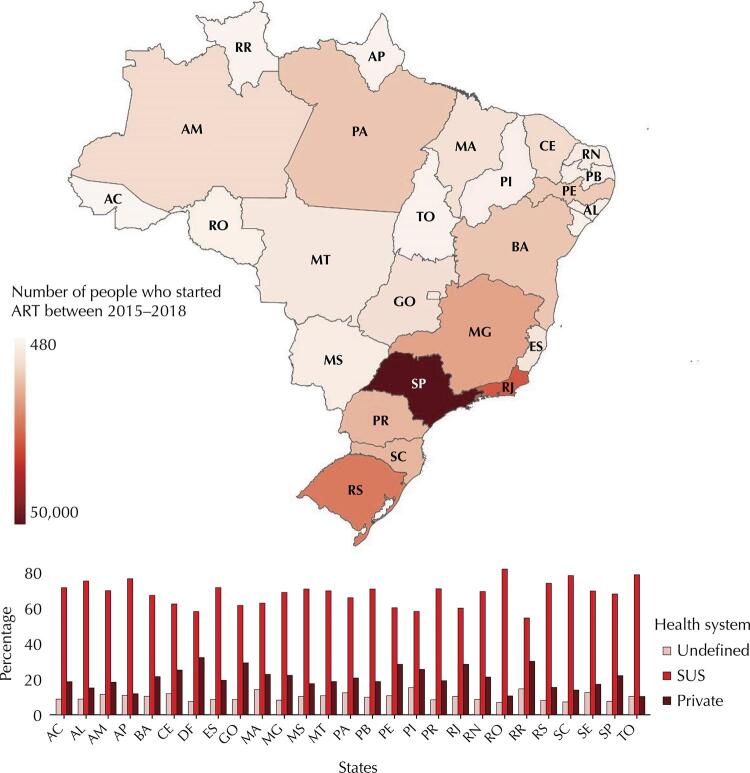
Distribution of people aged 15 and over who started ART between 2015 and 2018, according to the type of system where they receive HIV care. Qualiaids-BR cohort, 2021.

Despite the absolute number of people being concentrated in the Southeast (SP, 50,120; RJ, 27,898; MG, 16,266; ES, 4,878) and South (RS, 22,402; SC, 13,278; PR, 13,342) regions, the proportion of people followed-up at SUS facilities varied across the states with the highest proportions in Rondônia (RO, 82.4%) and Tocantins (TO, 80.2%) and the lowest proportions in Roraima (RR, 59.7%) and Distrito Federal (DF, 58.8%). Figure 2 presents the distribution according to the states.

### Typology of SUS HIV Care Facilities for Clinical-Laboratory Follow-up

In the period studied, 1,302 SUS facilities followed-up people aged 15 years or more starting antiretroviral therapy. Among them, those that were classified by self-report in the Qualiaids survey totaled 949 facilities (72.9%). Another 353 (27.1%) were classified according to name, of which 93 (7.1%) were from the prison system. It was not possible to classify 53 (4.1%) facilities.

Among the people followed-up at SUS, 161,854 (98.3%) were followed-up in 1,156 (88.7%) outpatient or primary care facilities. Another 1,718 (1%) received care in the prison system, 431 (0.3%) were followed-up in facilities for which it was not possible to define the type, and another 664 (0.4%) were linked only to hospitalization.

Outpatient clinics followed-up 153,699 people (93.3%) in 769 (59.1%) facilities; 8,155 people (5%) received care in 387 (29.7%) primary care facilities ( Table ). Among primary care facilities, 190 (49.1%) had less than ten new patients in the studied period and another 162 (41.9%) had up to 50 new patients. Outpatient clinics follow-up a larger volume of patients: 55.2% (461) had between 51 and 500 new patients.

**Table:** Distribution of the different types of SUS healthcare facilities that provide follow-up for HIV, according to the number of people over 15 who started ART between 2015 and 2018. Qualiaids-BR Cohort, 2021.

Number of people who started ART	Outpatient clinic				Primary care			
	
	People		Facilities		People		Facilities	
			
	n	%	n	%	n	%	n	%
≤ 10	250	0.2	45	5.9	1,006	12.3	190	49.1
11–50	5532	3.6	178	23.1	3,169	38.9	162	41.9
51–100	12,615	8.2	170	22.1	1,400	17.2	19	4.9
101–500	65,148	42.4	291	37.8	2,580	31.6	16	4.1
501–1,000	45,358	29.5	69	9.0				
> 1,000	24,796	16.1	16	2.1				
**Total**	**153,699**	**100.0**	**769**	**100.0**	**8,155**	**100.0**	**387**	**100.0**

Source: Qualiaids-BR cohort, 2021.

## DISCUSSION

HIV care in Brazil was established based on the recognition of access to health as a right for all citizens, in the same movement that resulted, years later, in the creation of SUS in 1988^13^ .

This should be the first study that estimates the relative size of SUS in the clinical-laboratory follow-up of HIV based on data produced in the healthcare facilities. Previous estimates were based on the number of private health insurance plans contracted, released by the National Supplementary Health Agency^16 , 17^ .

The data allowed estimating that, in addition to maintaining an extensive network of free supply of antiretroviral medication for all people living with HIV with medical prescription, the teams of the SUS healthcare facilities are responsible for the clinical follow-up of the majority. The estimated proportion of 69% may be lower than the real one, considering that the observation period of the primary study was only four years, not allowing the attribution of 9% of the people included. It should also be noted that the “size” of the SUS – and of the private system – is evidently much larger. This and other estimates in the study are based only on the incidence of new cases that started treatment between 2015 and 2018, which disregards cases already followed-up and underestimates the proportion of people in the system. Both systems currently follow-up a total of 766,000 people^18^ .

The study shows heterogeneity in the organization of SUS healthcare facilities, already pointed out in previous studies^4 , 6^ . By working with patient data, this study brought more details to the heterogeneous profile of the network. “Outpatient clinic” type of facilities – which include different outpatient modalities, such as medical specialty outpatient clinics, specialized STI/HIV and viral hepatitis outpatient clinics, hospital outpatient clinics or outpatient clinics with specialized teams inserted in a primary health center – are more numerous and follow-up most people living with HIV. The outpatient clinic type contains all the facilities with more than 500 people who started antiretroviral therapy in the period 2015–2018. Part of these facilities, especially the large ones, are those implemented in the first decades of the epidemic, many of them in general and/or school hospitals.

The group of facilities of the primary care type (community health center, family health center) is probably of more recent implementation. The follow-up of antiretroviral therapy in primary care facilities is, like all other implementations of SUS services, a municipal responsibility. The federal and state instances can only encourage it or not. Thus, although federal administrations occasionally recommended it, as of 2014 the federal government explicitly encouraged it, through regulations and technical support programs^21^ . This movement seems to have resulted in the implementation of antiretroviral therapy follow-up in primary care facilities in some municipalities in the country. The “primary health-care” type includes, for the most part, small-volume patient facilities that, although relatively numerous, follow only a proportion of less than 5% of people who start antiretroviral therapy. Further studies are needed to further detail this organizational profile of HIV care in SUS, with the aim of better contributing to service implementation policies.

## CONCLUSION

This study was only possible because SUS maintains a national system of continuous registration of antiretroviral medication dispensing for all people followed-up in the public or private system. However, viral load test records, an international standard for monitoring the treatment of HIV infection, are restricted to those who are followed-up in the public system. Data from these two systems are systematically linked to notification and mortality databases, allowing the dissemination of epidemiological bulletins and clinical monitoring reports, synthesized in a panel of public access indicators disaggregated by municipality^24^ . The private system is not subject to any regulation regarding the transparency of HIV data, not even the disclosure of the number of people living with HIV assisted, which makes it difficult to estimate morbidity and follow-up.

This study has limitations. Estimates were based only on those aged 15 years and older who started treatment between 2015-2018 and were followed for up to four years. It is possible that the estimates do not correspond to the proportions of people followed, especially for older large facilities, which may be restricting the enrollment of new patients. The strict division of follow-up into the public or private system also ignores the people who use both systems/facilities (public-private mix)^25^ already pointed out in Brazilian studies^26^ . Furthermore, for a small proportion of facilities, the outpatient clinic/primary care split based on service name alone may not have correctly distinguished some facilities. Despite the limits, the first national profile of the organization of HIV care produced by the Qualiaids-BR Cohort study can inform the management of health systems as well as subsidize new analyses.

## References

[1] Greco DB, Simão M (2007). Brazilian policy of universal access to AIDS treatment: sustainability challenges and perspectives. AIDS.

[2] Basso CR, Negri B, Viana ALD (2002). O Sistema Único de Saúde em 10 anos de desafio.

[3] Castro MC, Massuda A, Almeida G, Menezes-Filho NA, Andrade MV, Noronha KVMS (2019). Brazil’s unified health system: the first 30 years and prospects for the future. Lancet.

[4] Melchior R, Nemes MIB, Basso CR, Castanheira ERL, Alves MTSSB, Buchala CM (2006). Avaliação da estrutura organizacional da assistência ambulatorial em HIV/Aids no Brasil. Rev Saude Publica.

[5] Nemes MIB, Alves AM, Loch AP (2016). Sistema de avaliação Qualiaids.

[6] Nemes MIB, Melchior R, Basso CR, Castanheira ERL, Alves MTSSB, Shaun C (2009). The variability and predictors of quality of AIDS care services in Brazil. BMC Health Serv Res.

[7] Nemes MIB, Alencar TMD, Basso CR, Castanheira ERL, Melchior R, Alves MTSSB (2013). Assessment of outpatient services for AIDS patients, Brazil: comparative study 2001/2007. Rev Saude Publica.

[8] Loch AP, Nemes MIB, Santos MA, Alves AM, Melchior R, Basso CR (2018). Avaliação dos serviços ambulatoriais de assistência a pessoas vivendo com HIV no Sistema Único de Saúde: estudo comparativo 2007/2010. Cad Saude Publica.

[9] Ministério da Saúde (BR), Secretaria de Vigilância em Saúde (2021). Panorama epidemiológico da coinfecção TB-HIV no Brasil 2020. Bol Epidemiol.

[10] Ministério da Saúde (BR), Secretaria de Vigilância em Saúde (2021). Bol Epidemiol HIV/Aids.

[11] Meireles MV, Pascom ARP, Duarte EC (2018). Factors associated with early virological response in HIV-infected individuals starting antiretroviral therapy in brazil (2014-2015): results from a large HIV surveillance cohort. J Acquir Immune Defic Syndr.

[12] Mangal TD, Meireles MV, Pascom ARP, Coelho RA, Benzaken AS, Hallett TB (2019). Determinants of survival of people living with HIV/AIDS on antiretroviral therapy in Brazil 2006–2015. BMC Infect Dis.

[13] Brasil, Constituição (1988) (2016). Constituição da República Federativa do Brasil de 1988.

[14] Nemes MIB, Castanheira ERL, Loch AP, Santos MA, Alves AM, Melchior R, Akerman M, Furtado JP (2016). Práticas de avaliação em saúde no Brasil – Diálogos.

[15] Nemes MIB, Scheffer M, Basthi A, Parker R, Terto V (2016). Mitos vs Realidade sobre a resposta brasileira à epidemia de HIV e AIDS em 2016.

[16] Ministério da Saúde (BR), Agência Nacional de Saúde Suplementar (2011). Informações em Saúde Suplementar: ANS TABNET.

[17] Pascom ARP, Meireles MV, Benzaken AS (2018). Sociodemographic determinants of attrition in the HIV continuum of care in Brazil, in 2016. Medicine.

[18] Ministério da Saúde (BR), Secretaria de Vigilância em Saúde, Departamento de Vigilância, Prevenção e Controle das Infecções Sexualmente Transmissíveis, do HIV/Aids e das Hepatites Virais (2021). Relatório de monitoramento clínico do HIV 2020.

[19] Nemes MIB, Castanheira ERL, Melchior R, Alves MTSSB, Basso CR (2004). Avaliação da qualidade da assistência no programa de AIDS: questões para a investigação em serviços de saúde no Brasil. Cad Saude Publica.

[20] Nemes MIB, Catanheira ERL, Santa Helena ET, Melchior R, Caraciolo JM, Basso CR (2009). Adesão ao tratamento, acesso e qualidade da assistência em Aids no Brasil. Rev Assoc Med Bras.

[21] Ministério da Saúde (BR), Secretaria de Vigilância em Saúde (2014). Cinco passos para a implementação do manejo da infecção pelo HIV na Atenção Básica: guia para gestores.

[22] Ministério da Saúde (BR), Secretaria de Vigilância em Saúde (2015). O manejo da infecção pelo HIV na Atenção Básica: manual para profissionais médicos.

[23] Melo ED, Maksud I, Agostni R (2018). Cuidado, HIV/Aids e atenção primária no Brasil: desafio para a atenção no Sistema Único de Saúde?. Rev Panam Salud Publica.

[24] Ministério da Saúde (BR), Secretaria de Vigilância em Saúde, Departamento de Doenças de Condições Crônicas e Infecções Sexualmente Transmissíveis (2016). Painel de Indicadores Epidemiológicos.

[25] Chernichovsky D (2000). The public-private mix in the modern health care system - concepts, issues, and policy options revisited. NBER Work Pap Ser.

[26] Santos IS, Ugá MAD, Porto SM (2008). O mix público-privado no Sistema de Saúde Brasileiro: financiamento, oferta e utilização de serviços de saúde. Cien Saude Colet.

[27] Ministério da Saúde (BR), Secretaria de Vigilância em Saúde, Departamento de Vigilância, Prevenção e Controle das Infecções Sexualmente Transmissíveis, do HIV/Aids e das Hepatites Virais (2018). Protocolo clínico e diretrizes terapêuticas para manejo da infecção pelo HIV em adultos.

