# Supplementary material for: Beyond access to medication: the role of SUS and the characteristics of HIV care in Brazil
Source: Rev Saude Publica. 2023 Aug 28;57:26. doi: 10.11606/s1518-8787.2023057004476 (PMC10118421; doi:10.11606/s1518-8787.2023057004476)
Supplement: Translation

# Translation: Para além do acesso ao medicamento: papel do SUS e perfil da assistência em HIV no Brasil

**Authors:** Ana Maroso Alves, Angélica Carreira dos Santos, Aline Kumow, Ana Paula Sayuri Sato, Ernani Tiaraju de Santa Helena, Maria Ines Battistella Nemes

**Affiliations:** I Brasil Universidade de São Paulo. Faculdade de Medicina. Departamento de Medicina Preventiva. São Paulo, SP, Brasil; II Brasil Universidade de São Paulo. Faculdade de Medicina. Departamento de Pediatria. São Paulo, SP, Brasil; III Brasil Universidade de São Paulo. Faculdade de Saúde Pública. Departamento de Epidemiologia. São Paulo, SP, Brasil; IV Brasil Universidade de São Paulo. Faculdade de Saúde Pública. Departamento de Saúde Pública. São Paulo, SP, Brasil; V Brasil Universidade Regional de Blumenau. Departamento de Medicina. Blumenau, SC, Brasil

**Keywords:** Fármacos Anti-HIV, provisão & distribuição, Qualidade, Acesso e Avaliação da Assistência à Saúde, Sistema Único de Saúde, Saúde Suplementar

## Abstract

**OBJETIVO:**

Estimar a composição público-privada da assistência em HIV no Brasil e o perfil organizacional da extensa rede de serviços públicos.

**MÉTODOS:**

Foram utilizados dados da Coorte Qualiaids-BR, que reúne dados dos sistemas nacionais de informações clínicas e laboratoriais de pessoas com 15 anos ou mais com primeira dispensação de terapia antirretroviral, entre 2015–2018, e informações dos serviços do SUS de acompanhamento clínico-laboratorial do HIV, produzidas pelo inquérito Qualiaids. O sistema de acompanhamento foi definido pelo número de exames de carga viral solicitados por algum serviço do SUS: acompanhamento no sistema privado – nenhum registro; acompanhamento no SUS – dois ou mais registros; acompanhamento indefinido – um registro. Os serviços do SUS foram caracterizados como ambulatórios, atenção básica e sistema prisional, segundo autoclassificação dos respondentes ao inquérito Qualiaids (72,9%); para os não respondentes (27,1%) a classificação baseou-se nos termos presentes nos nomes dos serviços.

**RESULTADOS:**

No período, 238.599 pessoas com 15 anos ou mais iniciaram a terapia antirretroviral no Brasil, das quais, 69% receberam acompanhamento no SUS, 21,7% no sistema privado e 9,3% tiveram o sistema indefinido. Entre os acompanhados no SUS, 93,4% foram atendidos em serviços do tipo ambulatório, 5% em serviços de atenção básica e 1% no sistema prisional.

**CONCLUSÃO:**

No Brasil o tratamento antirretroviral é fornecido exclusivamente pelo SUS, que também é responsável pelo acompanhamento clínico-laboratorial da terapia da maior parte das pessoas em serviços ambulatoriais. O estudo só foi possível porque o SUS mantêm registros e informações públicas acerca do acompanhamento em HIV. Não há nenhum dado disponível para o sistema privado.

## INTRODUÇÃO

Da introdução do AZT (zidovudina) em 1993 até a emergência dos potentes antirretrovirais atuais, o acesso livre e universal à terapia antirretroviral do HIV/aids (TARV) distinguiu internacionalmente a resposta brasileira à epidemia de aids^1^ .

A instituição do programa nacional de DST/aids, em 1986^1^ , ensejou a implantação de serviços hospitalares e ambulatoriais para atendimento de pessoas com aids, a maioria em estruturas pré-existentes do sistema público de saúde^2^ , que, em 1988, tornou-se oficialmente o Sistema Único de Saúde (SUS)^3^ . A modalidade de assistência clínica ambulatorial, inicialmente denominada como serviço de assistência especializada em DST/aids, foi implantada em vários estados do país. Com a disseminação da epidemia no país e a incorporação da terapia antirretroviral, que converteu a doença pelo HIV em condição crônica, o número de serviços ambulatoriais teve grande expansão: registros do Ministério da Saúde mostram que o número de serviços aumentou mais de 3.000% em 20 anos (de 33, em 1996^4^ , para 1.060, em 2016)^5^ .

Como a estrutura do SUS é descentralizada, a implantação de serviços tornou-se da competência dos municípios. Inquéritos seriados sobre a organização dos serviços mostraram que esse tipo de gestão resultou em um conjunto heterogêneo de serviços, com variável configuração administrativa (serviços de atenção básica, clínicas de especialidades, serviços especializados em IST/HIV, ambulatórios de hospitais) e volume de pacientes (de um até mais de mil)^6^ .

Desde o início da implantação do tratamento medicamentoso do HIV até hoje, o SUS é o único comprador e fornecedor de medicamentos da terapia antirretroviral no Brasil e a dispensação do tratamento é realizada pelos serviços do sistema público. Assim, as pessoas com prescrição médica de antirretroviral são registradas em algum serviço local do SUS para receber os medicamentos. Um sistema nacional registra continuamente todas as dispensações de terapia antirretroviral (SICLOM – Sistema de controle logístico de medicamentos). Além desse sistema, todos os exames de carga viral e CD4 realizados no SUS são registrados em um sistema de informações centralizado nacionalmente (SISCEL – Sistema de controle de exames laboratoriais). Não há registros públicos que permitam o monitoramento do acompanhamento assistencial por parte dos serviços privados, embora recomende-se que os pacientes do sistema privado exibam o resultado do exame de carga viral mais recente no momento da dispensação de medicamentos.

Não há, até o momento, estudos que tracem o perfil organizativo nacional da assistência em HIV do país. Visando contribuir para o aprimoramento das políticas de implementação de serviços, este estudo objetiva estimar a composição público-privada da assistência em HIV no Brasil bem como o perfil organizacional da extensa rede de serviços públicos.

## MÉTODOS

### Fonte de Dados e População

O estudo utiliza dados secundários e anonimizados do projeto de pesquisa em andamento “Coorte Qualiaids-BR”, aprovado por Comitê de Ética em Pesquisa com seres humanos (CAEE: 27659220.3.0000.0065), que reúne informações das pessoas com 15 anos ou mais com registro no SICLOM de primeira dispensação de antirretroviral entre 1º de janeiro de 2015 e 31 de dezembro de 2018, e as informações dos serviços ambulatoriais de assistência em HIV do SUS.

A base de dados da coorte Qualiaids-BR foi construída a partir de dois bancos de dados: 1) Banco das pessoas em terapia antirretroviral com dados clínicos e sociodemográficos relacionados individualmente via um algoritmo probabilístico, já validado e utilizado rotineiramente nos boletins epidemiológicos do Ministério da Saúde^9 , 10^ e em publicações da área^11 , 12^ . O banco, produzido anualmente pelo ministério, relaciona para cada uma das pessoas em terapia antirretroviral os dados provenientes dos sistemas de informações do SUS – SICLOM, SISCEL, SINAN (Sistema de informação sobre agravos de notificação) e SIM (Sistema de informações sobre mortalidade); 2) Banco de dados dos serviços provenientes da resposta do inquérito Qualiaids 2016/17 sobre a organização dos serviços do SUS que prescrevem medicamentos antirretroviral^5^ . O relacionamento direto entre os dois bancos se baseou nos dados de localização (CEP, logradouro) e de nomes dos serviços ( Figura 1 ).

**Figura 1:** Fontes de dados e processo de construção da base de Coorte Qualiaids-BR.

### Definição do Sistema de Saúde Predominante de Seguimento Clínico

Para definir o sistema de saúde no qual a pessoa realiza o acompanhamento clínico-laboratorial do tratamento da infecção pelo HIV, foram considerados todos os exames de carga viral solicitados ao longo do tratamento e a data de início da terapia antirretroviral, de acordo com os critérios sintetizados no Quadro 1 .

**Quadro 1:** Definição do sistema predominante de acompanhamento, segundo número de registros de exames de carga viral (CV) e início de terapia antirretroviral (TARV). Coorte Qualiaids-BR, 2021.

Sistema predominante de acompanhamento	Definição
SUS	**Pessoas com dois ou mais registros de CV Pessoas com mais de um registro de CV** – perda de seguimento^a^em até 66 dias^b^do início de TARV
Indefinido	**Pessoas sem registro de CV** – perda de seguimento em até 66 dias do início de TARV **Pessoas com um registro de CV** – perda de seguimento após 66 dias do início de TARV **Pessoas sem registro ou com um registro de CV** – com início de TARV após 26/10/2018^c^
Privado	**Pessoas sem registro ou com um registro de CV** – com início de TARV anterior a 26/10/2018 **Pessoas sem registro** – com perda de seguimento após 66 dias do início TARV

^a^A perda de seguimento foi definida como óbito ou abandono definitivo de TARV (que considera se a última retirada de medicamento foi registrada até 100 dias antes da data final da coorte: 31/12/2018), em consonância com o critério definido pelo Ministério da Saúde^17^.

^b^O intervalo de 66 dias baseia-se na definição do Protocolo de Diretrizes Clínicas e Terapêuticas^27^de que, para pessoas em início de TARV, o intervalo entre exames de CV deve ser de até 56 dias. A esse intervalo foi acrescido outros 10 dias, considerando finais de semanas e feriados.

^c^A data foi definida considerando-se com o final da coorte (31/12/2018) e subtraída de 66 dias.

Para as pessoas cujo acompanhamento é predominantemente realizado em serviços do SUS, o serviço de acompanhamento foi definido como aquele que solicitou os exames de carga viral. Para aqueles que tiveram exames solicitados por mais de um serviço, foi considerado aquele que solicitou mais exames e, quando o número de exames solicitados foi igual, foi considerado aquele em que o paciente passou mais tempo vinculado. O tempo de vínculo ao serviço foi calculado pela diferença entre a primeira e a última solicitação de carga viral no determinado serviço.

Para caracterizar o tipo de serviço, foi utilizada a resposta do serviço a uma pergunta estruturada de resposta única que consta no inquérito Qualiaids-2016/2017^5^ . As alternativas descrevem os possíveis tipos administrativos, e foram agregadas em dois tipos: (1) Ambulatório (ambulatório exclusivo de assistência especializada a pacientes com HIV/aids, ambulatório de DST e hepatites virais; Ambulatório especializado em moléstias infecciosas; Equipe de assistência especializada inserida em um serviço de atenção básica; Ambulatório de assistência a várias especialidades e ambulatório de hospital); (2) Serviço de atenção básica (unidade básica de saúde, centro de saúde, saúde da família). Para os serviços que não responderam ao inquérito, a atribuição foi feita a partir da busca de termos presentes no nome de registro do serviço no SISCEL, conforme Quadro 2 .

**Quadro 2:** Classificação dos tipos de serviços a partir de termos presentes no nome do serviço. Coorte Qualiaids-BR, 2015–2018.

Tipo de serviço	Nome/termos
Ambulatório	AME (Ambulatório Médico de Especialidades); Centro de atendimento de doenças infectocontagiosas; Centro especializado em doenças infectocontagiosas; SAE (Serviço de atendimento especializado); Centro de Especialidades; Centro de Diagnóstico e Tratamento; Centro de Orientação e Aconselhamento; Centro de Referência; Centro de Testagem e Aconselhamento; Centro Especializado de Assistência à Saúde; Centro Estadual Regional de Média e Alta Complexidade; Centro Regional de Especialidades; Complexo Ambulatorial; Policlínica e Unidade de referência
Atenção básica	Centro de Saúde; Centro Municipal de Saúde (CMS); Clínica da Família (CF); Centro de Saúde (CS); Estratégia de Saúde da Família (ESF); Núcleo de apoio à Saúde da Família (NASF); Posto de Saúde; Unidade Básica de Saúde (UBS) e Unidade de Saúde
Serviço do sistema prisional	Casa de Custódia; Penitenciária; Sistema Penitenciário; Presídio; Centro de atendimento à mulher presa; ‘Detenção’ e ‘Penal’
Não foi possível atribuir	Núcleo de Apoio à Saúde; Unidade de Vigilância Epidemiológica; ‘Fundação’; Regional de Saúde; Associação; Casa de Apoio; Departamento de Ações de Saúde; Diretoria Regional de Saúde e Consórcio Intermunicipal

### Análise de Dados

Foram descritas as distribuições absolutas e relativas das pessoas da Coorte Qualiaids-BR, segundo o sistema predominante de acompanhamento para o HIV (SUS, privado ou indefinido), estado geográfico brasileiro e tipologia dos serviços do SUS (ambulatório, atenção básica e sistema prisional).

## RESULTADOS

### Sistema de Acompanhamento Clínico-Laboratorial

No período analisado, 238.599 pessoas com 15 anos ou mais iniciaram a terapia antirretroviral no Brasil. Desse total, 164.667 (69%) tiveram acompanhamento clínico-laboratorial em serviços do SUS e 51.879 (21,7%) foram acompanhadas em serviços privados. Não foi possível atribuir o sistema predominante de acompanhamento para 22.053 (9,3%) pessoas. Entre as pessoas assistidas no SUS, 132.086 (80,2%) tiveram todos os exames solicitados pelo mesmo serviço e 32.581 (19,8%) por mais de um serviço.

Apesar do número absoluto de pessoas estar concentrado nas regiões sudeste (SP, 50.120; RJ, 27.898; MG, 16.266; ES, 4.878) e sul (RS, 22.402; SC, 13.278; PR, 13.342), a proporção de pessoas acompanhadas em serviços do SUS variou entre os estados com proporções maiores em Rondônia (RO, 82,4%) e Tocantins (TO, 80,2%) e as menores proporções em Roraima (RR, 59,7%) e Distrito Federal (DF, 58,8%). A Figura 2 apresenta a distribuição segundo os estados.

**Figura 2:** Distribuição das pessoas com 15 anos ou mais que iniciaram TARV entre 2015 e 2018, segundo tipo de sistema onde recebem assistência para o HIV. Coorte Qualiaids-BR, 2021.

### Tipologia dos Serviços do SUS de Acompanhamento Clínico-Laboratorial

No período destacado para a pesquisa, 1.302 serviços do SUS acompanharam pessoas com 15 anos ou mais em início de terapia antirretroviral. Entre eles, aqueles que foram classificados por autorrelato no inquérito Qualiaids somaram 949 serviços (72,9%). Outros 353 (27,1%) foram classificados segundo o nome, dos quais 93 (7,1%) eram do sistema prisional. Não foi possível classificar 53 (4,1%) serviços.

Entre as pessoas acompanhadas no SUS, 161.854 (98,3%) foram acompanhadas em 1.156 (88,7%) serviços do tipo ambulatório ou atenção básica. Outras 1.718 (1%) foram assistidas no sistema prisional, 431 (0,3%) acompanhamentos em serviços para os quais não foi possível definir o tipo e outros 664 (0,4%) foram vinculadas apenas a internação hospitalar.

Os serviços do tipo ambulatório acompanharam 153.699 pessoas (93,3%) em 769 (59,1%) serviços; 8.155 pessoas (5%) foram assistidas em 387 (29,7%) serviços de Atenção Básica ( Tabela ). Entre os serviços do tipo atenção básica, 190 (49,1%) tiveram menos de dez pacientes novos no período estudado e outros 162 (41,9%) tinham até 50 pacientes novos. Os serviços do tipo ambulatório atendem um volume maior de pacientes: 55,2% (461) tiveram entre 51 e 500 pacientes novos.

**Tabela:** Distribuição dos diferentes tipos de serviços do SUS que fazem acompanhamento para o HIV, segundo número de pessoas maiores de 15 anos que iniciaram TARV entre 2015 e 2018. Coorte Qualiaids-BR, 2021.

Número de pessoas que iniciaram TARV	Ambulatório				Atenção básica			
	
	Pessoas		Serviços		Pessoas		Serviços	
			
	n	%	n	%	n	%	n	%
≤ 10	250	0,2	45	5,9	1.006	12,3	190	49,1
11–50	5532	3,6	178	23,1	3.169	38,9	162	41,9
51–100	12.615	8,2	170	22,1	1.400	17,2	19	4,9
101–500	65.148	42,4	291	37,8	2.580	31,6	16	4,1
501–1.000	45.358	29,5	69	9,0				
> 1.000	24.796	16,1	16	2,1				
**Total**	**153.699**	**100,0**	**769**	**100,0**	**8.155**	**100,0**	**387**	**100,0**

Fonte: Coorte Qualiaids-BR, 2021.

## DISCUSSÃO

A assistência em HIV no Brasil se estabeleceu com base no reconhecimento do acesso à saúde como um direito de todos os cidadãos, no mesmo movimento que resultou, anos mais tarde, na criação do SUS em 1988^13^ .

Este deve ser o primeiro estudo que estima o peso relativo do SUS no acompanhamento clínico-laboratorial do HIV com base em dados produzidos nos serviços. Estimativas anteriores foram baseadas no número de planos de saúde contratados, divulgados pela Agência Nacional de Saúde Suplementar^16 , 17^ .

Os dados permitiram estimar que, para além de manter uma extensa rede de fornecimento gratuito de medicação antirretroviral para todas as pessoas que convivem com HIV com prescrição médica, as equipes dos serviços do SUS são responsáveis pelo seguimento clínico da maioria. A proporção estimada de 69% pode ser menor do que a real, considerando que o período de observação do estudo primário foi de apenas quatro anos, não permitindo a atribuição de 9% das pessoas incluídas. Ressalte-se ainda que o “tamanho” do SUS – e do sistema privado – é, evidentemente, muito maior. Essa e as demais estimativas do estudo se baseiam apenas na incidência de novos casos que iniciaram o tratamento entre 2015 e 2018, o que desconsidera os casos já acompanhados e subestima a proporção de pessoas no sistema. Ambos os sistemas acompanham hoje um total de 766 mil pessoas^18^ .

O estudo mostra a heterogeneidade na organização dos serviços do SUS, já apontada em estudos anteriores^4 , 6^ . Ao trabalhar com dados dos pacientes, este estudo trouxe mais detalhes ao perfil heterogêneo da rede. Os serviços do tipo “ambulatório” – que incluem diferentes modalidades ambulatoriais, como ambulatórios de especialidades médicas, ambulatórios especializados em IST/HIV e hepatites virais, ambulatórios de hospitais ou ambulatórios com equipes especializadas inseridas em uma unidade básica de saúde são mais numerosos e acompanham a maioria das pessoas vivendo com HIV. O tipo ambulatório contém todos os serviços com mais de 500 pessoas que iniciaram a terapia antirretroviral no período 2015–2018. Parte desses serviços, especialmente os de grande porte, são os implantados nas primeiras décadas da epidemia, muitos deles em hospitais gerais e/ou de ensino.

O grupo de serviços do tipo atenção básica (unidade básica de saúde, unidades de saúde da família, centro de saúde) é, provavelmente, de implantação mais recente. O acompanhamento da terapia antirretroviral em serviços de atenção básica é, como todas as demais implantações de serviços do SUS, da competência municipal. As instâncias federal e estadual podem, apenas, estimulá-la ou não. Assim, embora gestões federais a tenham eventualmente recomendado, a partir de 2014 o governo federal incentivou-a explicitamente, por meio de normativas e programas de apoio técnico^21^ . Esse movimento parece ter redundado na implantação do acompanhamento da terapia antirretroviral em serviços de atenção básica em alguns municípios do país. O tipo “atenção básica à saúde” inclui, em sua maioria, serviços de pequeno volume de pacientes que, embora relativamente numerosos, acompanham apenas uma proporção de menos de 5% das pessoas que iniciaram a terapia antirretroviral. São necessários novos estudos para maior detalhamento desse perfil organizativo da assistência em HIV do SUS, com objetivo de melhor contribuir com as políticas de implantação de serviços.

## CONCLUSÃO

Este estudo só foi possível porque o SUS mantém um sistema nacional de registro contínuo das dispensações de medicamentos antirretrovirais para todas as pessoas acompanhadas no sistema público ou privado. Porém, os registros do exame de carga viral, padrão internacional para o acompanhamento do tratamento da infecção pelo HIV, se restringem aos que são acompanhados no sistema público. Os dados desses dois sistemas são sistematicamente relacionados com os de notificação e mortalidade, permitindo a divulgação de boletins epidemiológicos e relatórios de monitoramento clínico, sintetizados em painel de indicadores de acesso público desagregados por municípios^24^ . O sistema privado não é submetido a nenhuma normativa de transparência de dados sobre HIV, nem mesmo o da divulgação do número de pessoas vivendo com HIV atendidas, o que dificulta as estimativas de morbidade e acompanhamento.

Este estudo tem limitações. As estimativas se basearam apenas em maiores de 15 anos que iniciaram tratamento entre 2015–2018 e foram seguidos por um período de até quatro anos. É possível que as estimativas não correspondam às proporções de pessoas acompanhadas, especialmente para serviços de grande porte mais antigos, que podem estar restringindo a inscrição de novos pacientes. A divisão estrita do acompanhamento no sistema público ou privado também ignora as pessoas que utilizam ambos os sistemas/serviços (mix público-privado)^25^ já apontado em estudos brasileiros^26^ . Além disso, para uma pequena proporção dos serviços, a divisão ambulatório/atenção básica baseada apenas no nome do serviço pode não ter distinguido corretamente alguns serviços. Em que pesem os limites, o primeiro retrato nacional da organização da assistência em HIV produzido pelo estudo Coorte Qualiaids-BR pode informar a gestão dos sistemas de saúde bem como subsidiar novas análises.
